# Metal artifact reduction in patients with total hip replacements: evaluation of clinical photon counting CT using virtual monoenergetic images

**DOI:** 10.1007/s00330-023-09879-4

**Published:** 2023-07-12

**Authors:** Julian Schreck, Kai Roman Laukamp, Julius Henning Niehoff, Arwed Elias Michael, Jan Boriesosdick, Matthias Michael Wöltjen, Jan Robert Kröger, Robert P. Reimer, Jan-Peter Grunz, Jan Borggrefe, Simon Lennartz

**Affiliations:** 1 Department of Radiology, Neuroradiology and Nuclear Medicine, Johannes Wesling University Hospital, Ruhr University Bochum, 44801 Bochum, Germany; 2 Institute for Diagnostic and Interventional Radiology, Faculty of Medicine and University Hospital Cologne, University of Cologne, 50937 Cologne, Germany; 3 Department of Diagnostic and Interventional Radiology, University Hospital Würzburg, Oberdürrbacher Straße 6, 97080 Würzburg, Germany; 4 Institute for Diagnostic and Interventional Radiology, University Hospital Cologne, Kerpener Straße 62, 50937 Cologne, Germany

**Keywords:** Multidetector computed tomography, Artifacts, Abdomen, Pelvis

## Abstract

**Objectives:**

To investigate photon-counting CT (PCCT)–derived virtual monoenergetic images (VMI) for artifact reduction in patients with unilateral total hip replacements (THR).

**Methods:**

Forty-two patients with THR and portal-venous phase PCCT of the abdomen and pelvis were retrospectively included. For the quantitative analysis, region of interest (ROI)–based measurements of hypodense and hyperdense artifacts, as well as of artifact-impaired bone and the urinary bladder, were conducted, and corrected attenuation and image noise were calculated as the difference of attenuation and noise between artifact-impaired and normal tissue. Two radiologists qualitatively evaluated artifact extent, bone assessment, organ assessment, and iliac vessel assessment using 5-point Likert scales.

**Results:**

VMI_110keV_ yielded a significant reduction of hypo- and hyperdense artifacts compared to conventional polyenergetic images (CI) and the corrected attenuation closest to 0, indicating best possible artifact reduction (hypodense artifacts: CI: 237.8 ± 71.4 HU, VMI_110keV_: 8.5 ± 122.5 HU; *p* < 0.05; hyperdense artifacts: CI: 240.6 ± 40.8 HU vs. VMI_110keV_: 13.0 ± 110.4 HU; *p* < 0.05). VMI_110keV_ concordantly provided best artifact reduction in the bone and bladder as well as the lowest corrected image noise. In the qualitative assessment, VMI_110keV_ received the best ratings for artifact extent (CI: 2 (1–3), VMI_110keV_: 3 (2–4); *p* < 0.05) and bone assessment (CI: 3 (1–4), VMI_110keV_: 4 (2–5); *p* < 0.05), whereas organ and iliac vessel assessments were rated highest in CI and VMI_70keV_.

**Conclusions:**

PCCT-derived VMI effectively reduce artifacts from THR and thereby improve assessability of circumjacent bone tissue. VMI_110keV_ yielded optimal artifact reduction without overcorrection, yet organ and vessel assessments at that energy level and higher were impaired by loss of contrast.

**Clinical relevance statement:**

PCCT-enabled artifact reduction is a feasible method for improving assessability of the pelvis in patients with total hip replacements at clinical routine imaging.

**Key Points:**

*• Photon-counting CT-derived virtual monoenergetic images at 110 keV yielded best reduction of hyper- and hypodense artifacts, whereas higher energy levels resulted in artifact overcorrection.*

*• The qualitative artifact extent was reduced best in virtual monoenergetic images at 110 keV, facilitating an improved assessment of the circumjacent bone.*

*• Despite significant artifact reduction, assessment of pelvic organs as well as vessels did not profit from energy levels higher than 70 keV, due to the decline in image contrast.*

## Introduction

The prevalence of total hip replacements (THR) in the general population of the USA is about 0.8%, equivalent to 1.4 million people, with about 5% of the elderly population having undergone THR [1]. In computed tomography (CT), THR can impair the diagnostic assessment due to metal artifacts caused by photon starvation effects and beam hardening [2, 3]. CT metal artifacts induced by THR are commonly more severe than artifacts from smaller prostheses and may significantly hamper the evaluation particularly of circumjacent bone. This is critical, as a substantial proportion of patients with THR requires revision due to loosening, infection, dislocation, or osteolysis [4, 5]. Moreover, the assessability of the lower abdomen and pelvis can be impaired, e.g., at the assessment of suspected cancer or infection.

Modern developments in CT technology have contributed to reducing metal artifacts of THR, notably dual-energy CT (DECT) derived virtual monoenergetic images (VMI) at higher photon energies and metal artifact reduction (MAR) algorithms [6–10].

With the introduction of photon-counting CT (PCCT) into clinical routine, a new technology with artifact reduction capabilities has become available. The cadmium-telluride crystal photon-counting detector differs in its mode of operation from the conventional energy-integrating detector (EID) as X-rays are directly transformed into an electric signal without using any intermediate light conversion step [11]. In PCCT, photons are absorbed by a semiconductor photo-anode, generating positive and negative charges and finally generating an electric signal. Due to their technical setup, PCCT detectors yield a more sensitive and higher resolved detection of X-ray photons as well a higher accuracy in determining photon energies. The associated potential advantages of PCCT are manifold and currently under investigation, including improved radiation dose efficiency and higher spatial resolution [12–15]. A previous study that was performed with a preclinical PCCT system elucidated the metal artifact reduction capabilities of this technology and concluded that high-energy thresholding may allow for a more efficient artifact reduction compared to EID-CT [16]. However, to the best of our knowledge, there are no studies yet to investigate the PCCT-enabled metal artifact reduction using the clinical system in THR, which appears as one of the most important use cases for metal artifact reduction.

Therefore, the purpose of this study was to examine PCCT-enabled artifact reduction in patients with THR and to determine VMI settings facilitating an optimal qualitative diagnostic assessment.

## Material and methods

### Patients

This was an IRB-approved, retrospective study for which the requirement to obtain written consent was waived. The imaging database of our hospital was searched for patients who were scanned between September 2021 and February 2022 on a clinical PCCT (NAEOTOM Alpha, Siemens Healthineers) system using a portal venous phase abdominopelvic protocol and who had unilateral THR at the time of the examination. Cases with bilateral THP (*n* = 2) were excluded in order provide a homogenous dataset. No patient was excluded due to gender, weight, age, or further characteristics. Following inclusion and exclusion of patients, 42 patients with unilateral THR constituted the final cohort for this study.

### Image acquisition and reconstruction

All patients included underwent a portal venous phase examination of the abdomen and pelvis on a clinical PCCT (NAEOTOM Alpha, software version Syngo CT VA40, Siemens Healthineers).

Examinations were performed for different clinical indications, as indicated in the “Results” section.

All scans were performed in supine position with scan parameters as follows: tube voltage of 120 kVp, detector configuration of 144 × 0.4 mm with automatic tube current modulation. Images were acquired at an image quality (IQ) level of 170 and a pitch of 0.8. The image quality level (IQ level) represents quality reference milliampere-seconds (mAs) corrected according to detector and CT geometry-specific properties. Depending on the present assessment protocol, gantry rotation time was 0.5 s.

Conventional polyenergetic images (CI) were reconstructed in 2-mm slice thickness with an increment of 1 mm using a body imaging kernel (Br36) and quantum iterative reconstruction (QIR) level 4/4. Virtual monoenergetic images (VMI) were acquired as spectral post-processing (SPP) data using a slice thickness of 0.6 mm with a quantum body imaging kernel (Qr36) and quantum iterative reconstruction (QIR) level 4/4. Quantum iterative reconstructions (QIR) are available from level 0 (no QIR) to level 4 (maximum QIR). CI and VMI images were then normalized to a slice thickness of 2 mm and an increment of 1 mm in Syngo.Via (VB60 version Siemens Healthineers). Furthermore, VMI at four different energy levels (70 keV, 110 keV, 150 keV, 190 keV) were reconstructed in axial view. The 40-keV increments of VMI were chosen based on the results of previous studies on VMI-based MAR [9, 17] as well as to avoid obscuring qualitative differences by increments that are too small.

### Quantitative image analysis

**Fig. 1:**
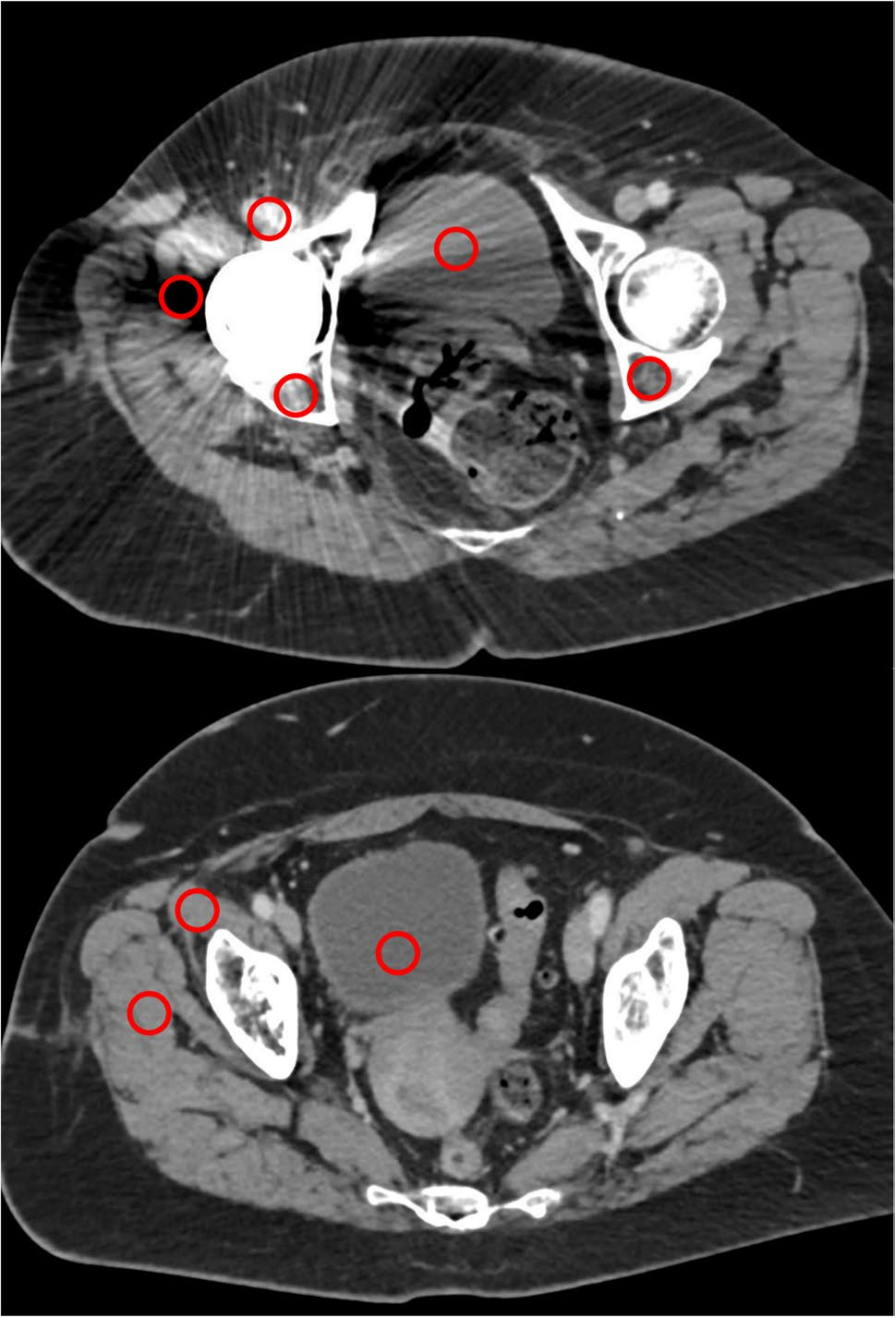
Region of interest (ROI) placement. ROIs were placed on polyenergetic images (CI) and subsequently copied and pasted on VMI (70 keV, 110 keV, 150 keV, 190 keV) in artifacts and corresponding contralateral artifact-free tissue. If the contralateral tissues were superimposed by artifacts, the nearest adjacent axial slice without artifacts was chosen to obtain measurements. Standard ROI size was set to 0.5 cm.^2^

The quantitative image analysis was executed by a radiologist with 2 years of experience in musculoskeletal imaging. Regions of interest (ROI) were placed on polyenergetic images (CI) and subsequently copied and pasted on VMI (70 keV, 110 keV, 150 keV, 190 keV) in the following locations, targeting the regions with the most pronounced artifacts: (i) hypodense and hyperdense artifacts adjacent to THR in the muscle (for each measurement in artifact-impaired tissue, a measurement in the corresponding artifact-free reference muscle was also applied), (ii) bone (acetabulum) with hyperdense artifact on the side of THR, contralateral bone (acetabulum) without artifact, and (iii) urinary bladder lumen (with artifact and without artifact). If the contralateral tissues were superimposed by artifacts, the nearest adjacent axial slice without artifacts was chosen to obtain reference measurements for artifact-free tissue. Standard ROI size was set to 0.5 cm^2^. Hounsfield unit (HU) of the encircled area was measured for the four defined energy levels and mean HU as well as their standard deviations for each ROI were recorded for further analysis. Figure 1 illustrates the ROI placement procedure.

To assess reduction of hypo- and hyperdense artifacts, the corrected attenuation was calculated as the difference of attenuation in tissue impaired by artifacts and artifact-free reference tissue; therefore, 0 HU indicated complete reduction of the artifact. Further, values higher than 0 HU in hypodense artifacts and values lower than 0 HU in hyperdense artifacts implied overcorrection of the initial artifacts [18]. The purpose of this method was to detect real artifacts and reduction hereof, while correcting for general changes along VMI energy levels that affect tissues regardless of artifact impairment. Furthermore, in a similar fashion, corrected image noise was calculated as the difference between image noise in artifact-impaired and artifact-free reference tissue [18, 19]. Corrected image noise can be considered to account for the lower image noise expected in VMI of higher energy levels.

### Qualitative image analysis

The qualitative image analysis was executed by two radiologists with 2 and 5 years of experience in musculoskeletal imaging who evaluated the following qualitative image criteria: extent of hyperdense/hypodense artifacts caused by THR, assessment of acetabular bone adjacent to the implant, and assessment of pelvic organs and the iliac vessels taking into account the interpretability of potential pathologies (e.g., fractures, tumors, thrombosis). The qualitative assessment was based on Likert scores which were assigned to the corresponding polyenergetic images (CI) and each individual VMI (70 keV, 110 keV, 150 keV, 190 keV). The radiologists both received detailed briefing prior to assessment. The readers were blinded to clinical and patient data. Further, they were blinded regarding the results of the quantitative analysis. The readers were given a complete set of images in each patient in the following order: CI, VMI at 70 keV, 110 keV, 150 keV, and 190 keV. For evaluation of artifact extent, the Likert scores were as follows: (5) artifacts are absent/almost absent, (4) minor artifacts, (3) moderate artifacts, (2) pronounced artifacts, (1) massive artifacts. For evaluation of different tissues, the following Likert scale was used: (1) insufficient, (2) restricted, (3) only marginally affected by minor artifacts, (4) not affected by minor streaks, (5) full diagnostic quality. Table 1 summarizes Likert scores and corresponding interpretations.

**Table 1:** Likert scores and corresponding interpretations used in the qualitative image evaluation

Extent of hypodense and hyperdense artifacts surrounding total hip prosthesis	(5) Artifacts are absent/almost absent
	(4) Minor artifacts
	(3) Moderate artifacts
	(2) Pronounced artifacts
	(1) Massive artifacts
Diagnostic assessment of the trabecular bone; considering evaluation of potential pathologies, e.g., loosening, fractures, and infections	(5) Fully diagnostic quality by no artifacts/almost no artifacts
	(4) Marginally affected diagnostic interpretability by minor streaks
	(3) Hampered diagnostic interpretability by moderate artifacts
	(2) Restricted diagnostic interpretability by strong artifacts
	(1) Insufficient diagnostic interpretability by metal artifacts
Diagnostic assessment of the pelvic organs; considering evaluation of potential pathologies, e.g., tumor, lymphadenopathy, and abscesses infections	(5) Fully diagnostic quality by no artifacts/almost no artifacts
	(4) Marginally affected diagnostic interpretability by minor streaks
	(3) Hampered diagnostic interpretability by moderate artifacts
	(2) Restricted diagnostic interpretability by strong artifacts
	(1) Insufficient diagnostic interpretability by metal artifacts
Diagnostic assessment of iliac vessels; considering evaluation of potential pathologies, e.g., stenosis and thrombosis	(5) Fully diagnostic quality by no artifacts/almost no artifacts
	(4) Marginally affected diagnostic interpretability by minor streaks
	(3) Hampered diagnostic interpretability by moderate artifacts
	(2) Restricted diagnostic interpretability by strong artifacts
	(1) Insufficient diagnostic interpretability by metal artifacts

### Statistical analysis

The statistical assessment was performed using software (JMP V14, SAS-Institute). The quantitative results are indicated as mean ± standard deviation. The qualitative results are presented as median and 10/90 percentile. The Shapiro–Wilk test was applied to test for normal distribution. The Wilcoxon test with Steel adjustment for multiple comparisons was used to test for any significant difference. The statistical significance was set to *p* < 0.05.

The inter-reader agreement was analyzed by application of Kendall’s *W* and interpreted as excellent (0.8–1.0), good (0.6−0.8), moderate (0.4−0.6), and poor (< 0.4), as suggested previously [20].

## Results

### Patients

Forty-two patients (23 women) with a mean age of 74.4 ± 9.2 years were included. CT scans of the abdomen and pelvis were performed in all patients in the venous phase for the following reasons: staging examinations or suspected cancer (*n* = 23), suspected abdominal infection (*n* = 11), trauma evaluation (*n* = 7), and postinterventional/operative evaluation (*n* = 1).

### Quantitative analysis

In all assessed tissues and in hypodense as well as hyperdense artifacts, VMI at 110 keV showed the optimal artifact reduction as indicated by a corrected attenuation close to 0 HU. Moreover, for hyper- and hypodense artifacts, corrected attenuation further decreased and increased, respectively, in VMI of higher energy levels (> 110 keV), indicating an overcorrection.

In muscle affected by hypodense artifacts, corrected attenuation significantly increased in VMI ≥ 110 keV compared to CI (CI: –237.8 ± 71.4, VMI_110keV_: 8.5 ± 122.5 HU, *p* < 0.05), whereas in hyperdense artifacts, corrected attenuation showed a significant decrease in VMI ≥ 110 keV compared to CI (CI: 240.6 ± 40.8, VMI_110keV_: 13.0 ± 110.4 HU, *p* < 0.05).

In concordance with the measurements in muscle tissue, corrected attenuation decreased in VMI ≥ 110 keV in artifact impaired bone (CI: 77.3 ± 101,8, VMI_110keV_: –18.0 ± 110.6 HU, *p* < 0.05), as well as bladder (CI: 45.3 ± 27.6, VMI_110keV_: –4.3 ± 34.8 HU, *p* < 0.05).

Corrected image noise significantly decreased in VMI ≥ 110 keV compared to CI in hypodense (CI: 47.3 ± 21.3, VMI_110keV_: 19.7 ± 22.8 HU, *p* < 0.05) and hyperdense artifacts (CI: 51.9 ± 22.5, VMI_110keV_: 27.5 ± 31.2 HU, *p* < 0.05). In artifact-impaired bone (CI: 22.6 ± 22.2, VMI_110keV_: 12.1 ± 16.0 HU, *p* > 0.05) and bladder (CI: 7.4 ± 9.6, VMI_110keV_: 3.6 ± 9.9 HU, *p* > 0.05), corrected image noise showed a decrease in VMI ≥ 110 keV; however, this was not statistically significant.

**Fig. 2:**
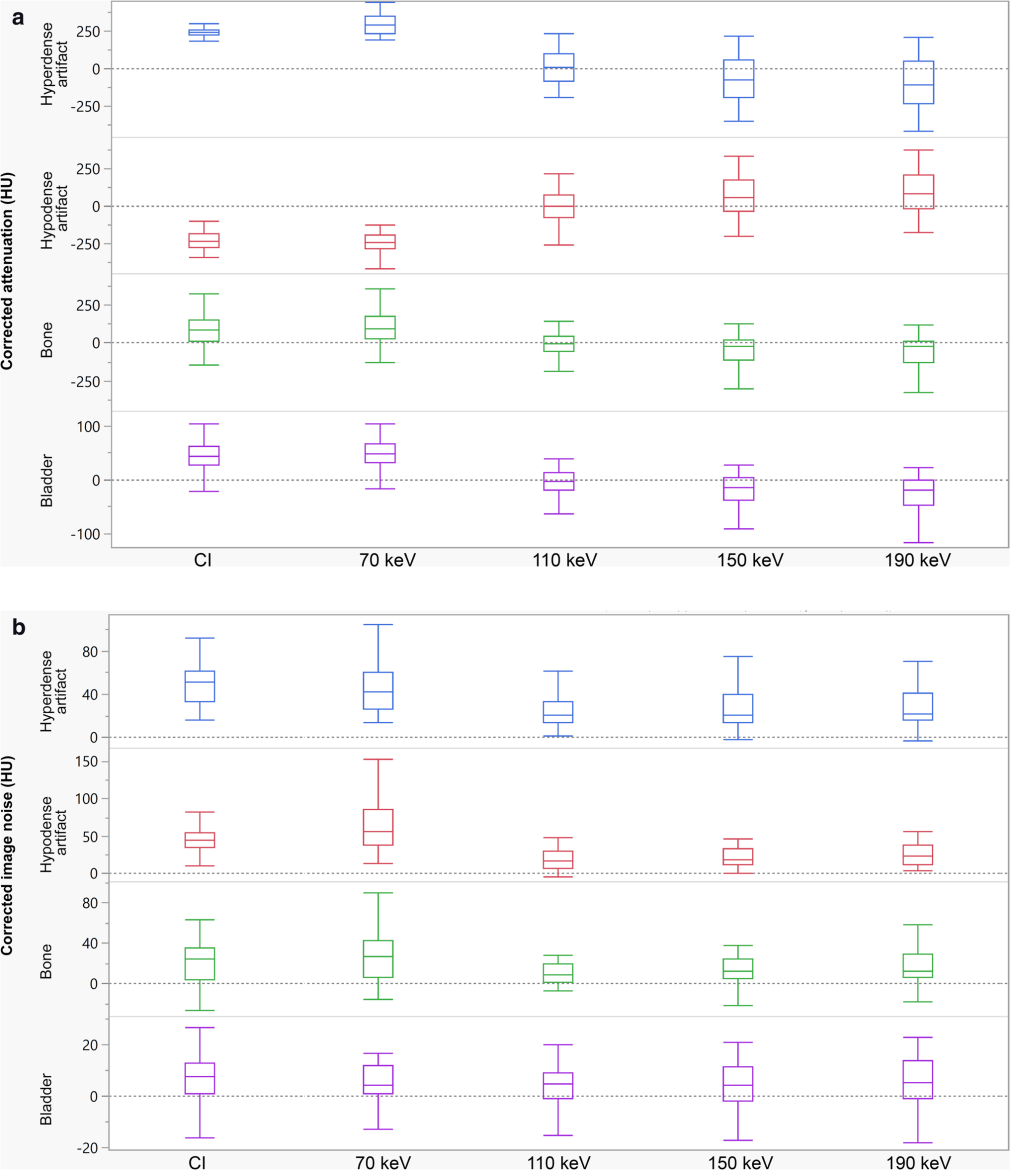
**a** Results of the quantitative image evaluation regarding corrected attenuation. Corrected image attenuation values were closest to 0 in virtual monoenergetic images at 110 keV (VMI_110keV_) for hyper- and hypodense artifacts in the muscle as well as in artifact-impaired bone and bladder. Corrected image attenuation values close to 0 indicate optimal artifact reduction. **b** Results of the quantitative image evaluation regarding corrected noise. Corrected image noise showed values closet to 0 in virtual monoenergetic images at 110 keV (VMI_110keV_) for hyper- and hypodense artifacts in the muscle as well as in artifact-impaired bone and bladder. Corrected image noise values close to 0 indicate optimal artifact reduction

The objective analysis results are displayed in Table 2 and Fig. 2.

**Table 2:** Results of the quantitative analysis of artifact reduction in hypo- and hyperdense artifacts in surrounding muscle, artifact impaired bone, and artifact impaired bladder

	Corrected attenuation				Corrected image noise			
	Hypodense artifacts	Hyperdense artifacts	Bone	Bladder	Hypodense artifacts	Hyperdense artifacts	Bone	Bladder
**T3D**	− 237.8 ± 71.4	240.6 ± 40.8	77.3 ± 101.8	45.3 ± 27.6	47.3 ± 21.3	51.9 ± 22.5	22.6 ± 22.2	7.4 ± 9.6
**VMI**								
70 keV	− 248.5 ± 70.5	298.6 ± 70.3	107 ± 108.2	54.1 ± 33.5	64.3 ± 38	45 ± 22.6	27.4 ± 25.5	6.8 ± 10.3
110 keV	8.5 ± 122.5	13 ± 110.4	− 18 ± 110.6	− 4.3 ± 34.8	19.7 ± 22.8	27.5 ± 31.2	12.1 ± 16	3.6 ± 9.9
150 keV	77.2 ± 153.7	− 63.6 ± 146.2	− 51.1 ± 142.1	− 19.7 ± 44.3	26.8 ± 31.4	32.4 ± 41.5	15.5 ± 18.3	4.2 ± 10.9
190 keV	102.7 ± 165.9	− 92.3 ± 159.7	− 63.4 ± 155.1	− 25.5 ± 48	31 ± 35.6	35.1 ± 45.3	17.6 ± 19.5	4.8 ± 11.6
***p values***								
T3D VS. 70 keV	> 0.05	< 0.05	> 0.05	> 0.05	> 0.05	> 0.05	> 0.05	> 0.05
T3D VS. 110 keV	< 0.05	< 0.05	< 0.05	< 0.05	< 0.05	< 0.05	> 0.05	> 0.05
T3D VS. 150 keV	< 0.05	< 0.05	< 0.05	< 0.05	< 0.05	< 0.05	> 0.05	> 0.05
T3D VS. 190 keV	< 0.05	< 0.05	< 0.05	< 0.05	< 0.05	< 0.05	> 0.05	> 0.05

Data is reported as mean ± standard deviation. *CI* conventional polyenergetic images, *VMI* virtual monoenergetic images. Bold indicates significant changes in HU compared to CI

### Qualitative analysis

**Fig. 3:**
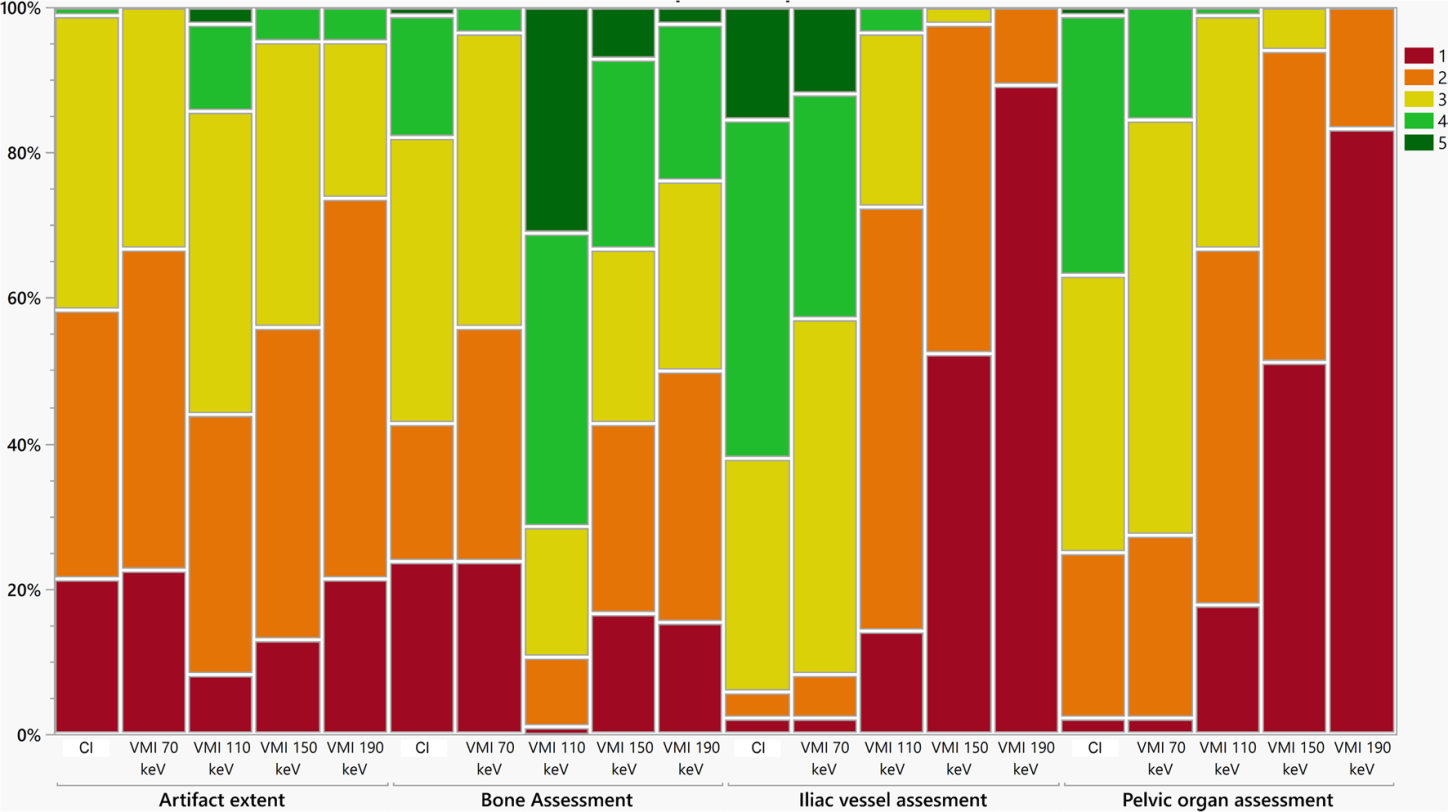
Color-coded stacked bar graphs showing the results of the qualitative assessment. Likert scores for artifact extent as well as bone assessment increased in virtual monoenergetic images (VMI) at 110 keV compared to polyenergetic (CI) images, whereas VMI at 110 keV were rated inferior compared to CI and VMI at 70 keV for iliac vessel and pelvic organ assessment

**Fig. 4:**
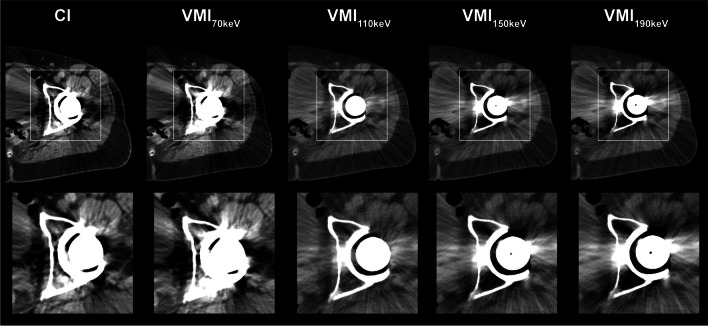
Image example of a patient with THR and predominantly hypodense artifacts. Optimal reduction of artifacts and assessment of circumjacent bone is provided in VMI at 110 keV, whereas VMI of higher energy levels show relevant overcorrection of the artifact

The extent of artifacts was significantly reduced in VMI at 110 keV compared to CI (CI: 2 (1–3), VMI_110keV_: 3 (2–4), *p* < 0.05). Further, bone assessment could be significantly improved using VMI at 110 keV compared to CI (CI: 3 (1–4), VMI_110keV_: 4 (2–5), *p* < 0.05). In contrast to the improved assessment of bone tissue, neither the diagnostic assessment of pelvic organs (CI: 3 (2–4), VMI_110keV_: 2 (1–3), *p* < 0.05) nor the assessment of iliac vessels benefited from using higher-energetic VMI (CI: 4 (3–5), VMI_110keV_: 2 (1–3), *p* < 0.05), whereas both criteria received comparable scores as CI when using VMI at 70 keV (*p* = 0.11 each). Inter-reader agreement in the qualitative image analysis was good for VMI_70keV_ (*W* = 0.77) and VMI_150keV_ (*W* = 0.77), and excellent for CI, VMI_110keV_ (*W* = 0.85) and VMI_190keV_ (*W* = 0.82). The results of the qualitative analysis are summarized in Table 3 and Fig. 3. Image examples for artifact reduction are shown in Fig. 4.

**Table 3:** Results of the qualitative analysis of artifact extent, bone assessment, pelvic organ assessment, and iliac vessel assessment

	Artifact extent	Bone assessment	Organ assessment	Iliac vessel assessment
**T3D**	2 (1–3)	3 (1–4)	3 (2–4)	4 (3–5)
**VMI**				
70 KEV	2 (1–3)	2 (1–3)	3 (2–4)	3 (3–5)
110 KEV	3 (2–4)	4 (2–5)	2 (1–3)	2 (1–3)
150 KEV	2 (1–3)	3 (1–4)	1 (1–2)	1 (1–2)
190 KEV	2 (1–3)	2.5 (1–4)	1 (1–2)	1 (1–2)
***p values***				
T3D VS. 70 keV	> 0.05	> 0.05	> 0.05	> 0.05
T3D VS. 110 keV	** < 0.05**	** < 0.05**	** < 0.05**	** < 0.05**
T3D VS. 150 keV	> 0.05	> 0.05	** < 0.05**	** < 0.05**
T3D VS. 190 keV	> 0.05	> 0.05	** < 0.05**	** < 0.05**

Data is reported as median with 10/90 percentile

*CI* conventional polyenergetic images, *VMI* virtual monoenergetic images

Bold indicates significant changes in scores compared to CI

## Discussion

In this study, we evaluated artifact reduction in patients with unilateral total hip replacements (THR) based on virtual monoenergetic images (VMI) reconstructed from abdominopelvic photon-counting CT (PCCT) examinations. We found that quantitative and qualitative artifact reduction was best in VMI at 110 keV, and that energy levels higher than that led to relevant overcorrection of artifacts. Artifact reduction facilitated by VMI_110keV_ allowed for a significant reduction of visually perceivable artifact impairment and hereby for an improved assessment of bone adjacent to the implant. The assessment of pelvic organs and vessels in VMI_70keV_ was comparable to conventional polyenergetic images (CI), yet ratings gradually decreased with increasing energy levels, most likely due to loss of iodine/soft tissue contrast.

Artifacts from THR are an issue of high clinical relevance, which is due to their high prevalence particularly in the elderly, as well as due to the fact that artifacts deriving from THR can be extensive. Such artifacts may severely hamper the assessment of the circumjacent pelvic bone, e.g., when assessing potential complications and determining the need for THR revision [5]. Furthermore, the evaluation of muscle and soft tissue components of the lower abdomen and pelvis may be impaired, which becomes relevant for oncologic assessment. Dual-energy CT (DECT) derived high-energy VMI have emerged as one important approach to reduce such artifacts.

Interestingly, in our study on PCCT-derived artifact reduction, we found a lower VMI energy level to be most effective in reducing artifacts from THR compared to previous studies. Whereas the optimal level we found was 110 keV, a VMI level of 140 keV was described as recommended in a previous study using a dual-layer DECT (dlDECT). Here, best artifact reduction was found between 140 and 200 keV; 140 keV was recommended as standard reconstruction in order to maintain a better soft tissue contrast [17]. Yue et al. assessed metal artifact reduction on a rapid kV switching DECT (rsDECT) and determined an energy level of 120–140 keV as optimal, whereas Huflage et al. suggested an energy level of 150 keV using a dual-source DECT (dsDECT) system [21]. In two phantom studies, similar keV values were proposed using dual-layer and dual-source DECT technology, proposing 130–150 keV as standard setting for VMI reconstructions in patients with THR [22, 23]. This suggests that the optimal keV value is not only influenced by the individual patient himself, the examined body part, the type of implant, and severity of artifacts [17, 24], but also by the manufacturer, the scanning technique (e.g., photon counting vs. dual energy), and/or the applied algorithm. Regardless of the scanner type, we agree with earlier studies that keV values should be adjusted in each patient individually to achieve optimal results [9, 19].

One of the key results of our study is the significantly improved assessment of the acetabular bone adjacent to the implant. Contrarily, the impaired assessment of the pelvic organs and vessels is in concordance with results of earlier investigations on DECT-based MAR [9, 19]. For evaluation of organs and vessels, artifact reduction algorithms could be an effective approach [17, 25], which were found to be particularly efficient in extensive artifact resulting from uni- and bilateral THR [26]. Combining metal artifact reduction algorithms with PCCT-derived VMI could therefore be one promising approach to tackle the shortfall of higher-energetic VMI in assessing pelvic organs. Moreover, advanced bin-selective imaging methods provided by PCCT might be another possible alternative.

Indicating artifact reduction, corrected image attenuation in hyper- and hypodense artifacts significantly decreased. Of note, standard deviation of corrected attenuation increased in VMI compared to CI. This effect is most likely explained by the fact that in the small ROIs placed into uncorrected artifacts, attenuation is elevated/decreased relatively homogenously, corresponding to a relatively lower standard deviation. In VMI, reduction of artifacts differs interindividually, e.g., in some patients optimal artifact reduction might be attained at 100 keV and in another patient at 115 keV. Therefore, despite the average reduction of artifacts indicated by corrected attenuations approximating 0, the values across patients become more heterogeneous, resulting in a relatively higher standard deviation when averaging over all patients that were included. This study has limitations that need to be acknowledged. First, it includes only a limited number of patients, which, in combination with the retrospective nature of our investigation, increases the risk for bias. Second, our results can only be applied to patients with unilateral THR, as during the inclusion period, only two patients with bilateral THR were examined, which were therefore not included in the analysis. As artifacts in these patients can be even more severe, the efficacy of PCCT-enabled metal artifact reduction in bilateral replacements should be examined in a dedicated study. Third, as the possibility to combine dedicated MAR algorithms with PCCT-derived VMI was not available at the time of our investigation, we were not able to address this question, which is to be further assessed in subsequent investigations. Fourth, it needs to be considered that in this retrospective analysis there was only limited data available on the type of implant and that the implants were heterogeneous; therefore, a dedicated subgroup analysis was not possible. Fifth, full blinding of readers was not feasible since images are distinguishable by their appearance. We also aimed to encourage readers to detect subtle differences between reconstructions; therefore, a full image set of one patient at a time was presented to the readers. Last, corrected attenuation and image noise were applied to detect real artifacts and reduction hereof, while correcting for general changes along VMI energy. We therefore calculated the difference of attenuation in tissue impaired by artifacts and artifact-free reference tissue [18, 19]. Still, it should be considered that comparing pre-implant and post-implant images, as Wellenberg et al. did in a cadaver study, might offer an even better ground truth [27].

To conclude, we found that PCCT-derived VMI are an effective means for reduction of artifacts from THR, allowing for a superior assessment of the acetabular bone, which may be advantageous when assessing potential complications or the necessity for revision. Future scientific efforts at the topic should explore the potential of combining MAR techniques with PCCT-derived VMI as well as advanced approaches such as energy binning.

## Abbreviations

CI: Polyenergetic images
DECT: Dual-energy CT
EID-CT: Energy-integrating detector CT
HU: Hounsfield units
keV: Kiloelectronvolt
PCCT: Photon-counting CT
ROI: Region of interest
THR: Total hip replacements
VMI: Virtual monoenergetic images

## Declarations

### Guarantor

The scientific guarantor of this publication is Jan Borggrefe.

### Statistics and biometry

No complex statistical methods were necessary.

### Methodology

- Retrospective
- Observational
- Performed at one institution
